# Recent household transmission of tuberculosis in England, 2010–2012: retrospective national cohort study combining epidemiological and molecular strain typing data

**DOI:** 10.1186/s12916-017-0864-y

**Published:** 2017-06-13

**Authors:** Maeve K. Lalor, Laura F. Anderson, Esther L. Hamblion, Andy Burkitt, Jennifer A. Davidson, Helen Maguire, Ibrahim Abubakar, H. Lucy Thomas

**Affiliations:** 1 TB Section, Centre for Infectious Disease Surveillance, National Infection Service, Public Health England, London, UK; 2 Institute for Global Health, University College London, London, UK; 3 Field Epidemiology Services, National Infection Service, Public Health England, London, UK; 4 Field Epidemiology Services, National Infection Service, Public Health England, Newcastle upon Tyne, UK

**Keywords:** Tuberculosis, Transmission, Molecular epidemiology, Household, MIRU-VNTR strain typing

## Abstract

**Background:**

We estimate the proportion of tuberculosis (TB) in England due to recent household transmission, identify factors associated with being a household transmitter, and investigate the impact that identification of a case has on time to treatment of subsequent cases.

**Methods:**

TB cases notified between 2010 and 2012 in England in the same household as another case were identified; 24 locus MIRU-VNTR strain typing (ST) was used to identify household cases with likely recent transmission. Treatment delay in index and subsequent cases was compared. Risk factors for being a household transmitter were identified in univariable and multivariable analyses.

**Results:**

Overall, 7.7% (1849/24,060) of TB cases lived in a household with another case. We estimate that 3.9% were due to recent household transmission. ST data was unavailable for 67% (1242) of household pairs. For those with ST data, 64% (386) had confirmed, 11% probable (66) and 25% (155) refuted household transmission. The median treatment delay was 65 days for index cases and 37 days for subsequent asymptomatic cases. Risk factors for being a household transmitter included being under 25 years old, UK-born with Black African, Indian or Pakistani ethnicity, or born in Somalia or Romania.

**Conclusions:**

This study has a number of implications for household TB contact tracing in low incidence countries, including the potential to reduce the diagnostic delay for subsequent household cases and the benefit of using ST to identify when to conduct source contact tracing outside the household. As 25% of TB cases in households had discordant strains, households with multiple TB cases do not necessarily represent household transmission. The additional fact that 25% of index cases within households only had extra-pulmonary TB demonstrates that, if household contact tracing is limited to pulmonary TB cases (as recently recommended in UK guidelines), additional cases of active TB in households will be missed. Our finding that no lineage of TB was associated with recent household transmission and with no increased transmissibility in the Beijing lineage compared to others, suggests that the lineage need not impact contact tracing efforts. Improvements in contact tracing have the potential to reduce transmission of TB in low incidence countries.

**Electronic supplementary material:**

The online version of this article (doi:10.1186/s12916-017-0864-y) contains supplementary material, which is available to authorized users.

## Background

Understanding whether tuberculosis (TB) cases are due to recent transmission or reactivation of remotely acquired latent infection would allow directed public health interventions to reduce TB. In England, a high proportion of TB cases are born abroad (73% in 2015), many of whom are likely to have been infected in their country of birth and reactivated in England [1].

Epidemiological information can help identify outbreaks. However, epidemiological data alone may either underestimate recent transmission (when there are no recognised epidemiological links) or overestimate recent transmission, especially in populations with an increased risk of TB, for example where there are a sizable number of persons who have immigrated from a high TB burden country, as evidenced by the utility of strain typing (ST) for refuting transmission [2].

Molecular ST data can provide information about whether cases are plausibly part of the same transmission chain. It can also be used to estimate the proportion of TB due to recent transmission, but has limitations [3]. ST data are only available for culture confirmed cases, and the currently used 24-loci Mycobacterial Interspersed Repetitive Unit-Variable Number Tandem Repeats (MIRU-VNTR) ST may not be discriminatory enough to adequately distinguish clusters [4]. ST data alone may overestimate recent transmission due to common strains circulating both in England and abroad [1]. Combining both epidemiological and ST data should provide better estimates of recent transmission.

Household contacts are at a higher risk of infection, and the household setting is an important reservoir for transmission [5, 6]. Studies have estimated the proportion of transmission within and outside the household in high-burden settings [3, 7–10], but not in low-burden settings. Household contact screening is key for TB control; enabling identification of active and latent TB cases in the household, and prompt treatment initiation and prevention of further transmission. Additionally, ‘inform and advise’ information should lead to earlier diagnosis and treatment for subsequent cases. In the UK, guidance on close contact screening has recently changed. The British Thoracic Society guidance in 2000 advised screening household/close contacts of pulmonary cases [11]. The National Institute for Health and Care Excellence (NICE) guidance was revised in 2016, from screening all household contacts of active TB cases irrespective of the site of disease [12], to only screening household contacts of pulmonary TB cases [13].

Our aim was to combine epidemiological and ST data to estimate the proportion of TB due to recent transmission in households in England, describe the characteristics of those in whom transmission has occurred, and identify the factors associated with being a household transmitter. Additionally, we investigated the impact that identification of a household TB case had on time to diagnosis and treatment of subsequent cases.

## Methods

TB cases notified to the Enhanced Tuberculosis Surveillance system in England between January 1, 2010, and December 31, 2012, were included in the analysis. All initial *M. tuberculosis* complex isolates were prospectively typed using 24-loci MIRU-VNTR ST, and data from isolates were probabilistically matched to TB case notifications [14].

### Identification of molecular links (ML)

*M. tuberculosis* complex isolates with indistinguishable MIRU-VNTR (at least one complete 24-loci typed, other isolates with at least 23-loci) were in the same cluster and defined as having ML. Cluster investigations were carried out following national guidance [15].

### Identification of household epidemiological links (HL)

TB cases were classified as having a HL to at least one other case if cases within the 3-year period were notified with the same address, independent of ST results, or if, following cluster investigation, two or more cases in the same household were identified and reported. For a small number of cases (*n* = 49), the specific address of the household was unknown and therefore these cases were excluded from the household level analysis.

### Classification of household transmission

Combining data on HL and ML, cases were classified as having been in a confirmed, probable, possible or refuted household transmission event.

**Fig. 1:**
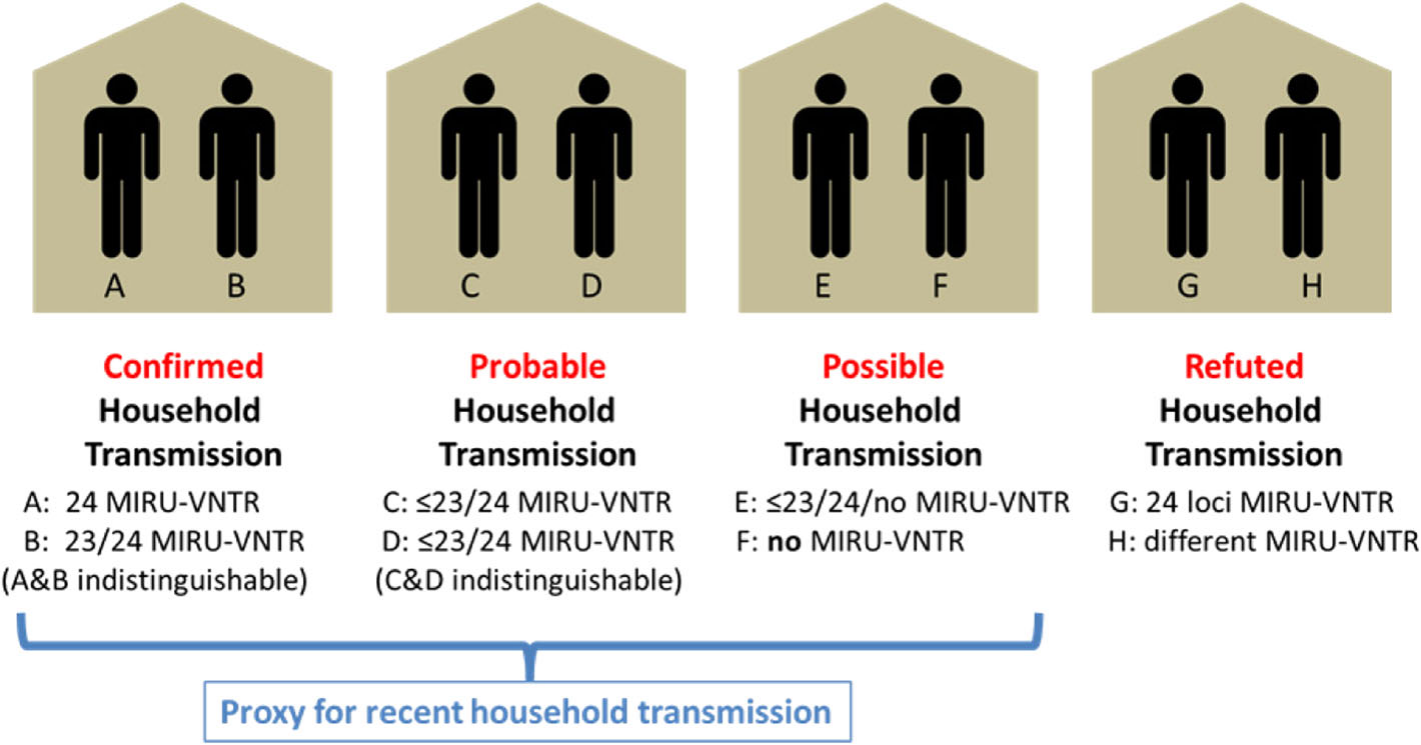
Classification of cases using epidemiological and strain typing data

We defined confirmed household transmission as two cases having both a HL and an ML; probable household transmission as two cases having a HL and an indistinguishable MIRU-VNTR, but where one/both cases were not typed to 23-loci; possible household transmission as two cases having a HL when one/both did not have a MIRU-VNTR profile; and refuted household transmission when two cases had a HL but with distinguishable MIRU-VNTR profiles (≥1 loci different) (Fig. 1).

TB cases with a HL without transmission refuted by ST (confirmed, probable and possible transmission) were used as a proxy for recent household transmission (Fig. 1). In a sensitivity analysis we tested whether the results were significantly different, defining recent household transmission as ‘confirmed and probable transmission’ or ‘confirmed, probable and possible transmission’.

The HL TB cases and associated ST were combined with data from the Enhanced Tuberculosis Surveillance System: patient demographic characteristics (age, sex, ethnicity, country of birth, address at notification), clinical characteristics (site of disease and date of symptom onset, presentation to healthcare, diagnosis, specimen collection and treatment start), and social risk factors (drug use, alcohol use, homelessness and imprisonment).

### Order of TB cases within household: defining the ‘index case’

To investigate the relationship between identification of a TB case in a household and time to diagnosis and treatment of subsequent cases, the cases in each household were ordered by date of first contact with healthcare (defined as the earliest date of presentation to healthcare, diagnosis, specimen collection or treatment start). The first case to have contact with healthcare, irrespective of site of disease, was defined as the household index case.

### Treatment delay and impact of household order on diagnosis of subsequent cases

Treatment delay was defined as the number of days between symptom onset and treatment start. The time between the index cases’ first contact with healthcare and treatment start for each subsequent case was calculated. Subsequent household cases (with a symptom onset date) were stratified according to whether or not they were symptomatic at the index case’s first contact with healthcare.

### Risk factors for being the transmitter in a household

To identify the probable direction of transmission of TB in a household and identify risk factors for being a probable transmitter, cases in households were ordered by date of symptom onset (or earliest contact with healthcare if no symptom onset date was available). The first pulmonary TB case in each household where there was at least one subsequent case was classified as the probable transmitter. Households with no pulmonary cases followed by subsequent cases did not have a probable transmitter identified. Univariable analysis to compare demographic and clinical characteristics of probable transmitters with all other TB cases (both those that were subsequent household cases and those who were not in a TB household) were conducted. A *P* value of less than 0.05 was considered to be statistically significant. A multivariable logistic regression to calculate adjusted odds ratios (aORs) for factors associated with household transmitters was conducted. A forward stepwise multivariable logistic regression was used with likelihood ratio tests assessed after each stepwise addition to the model. Analysis was conducted using Stata 13.1.

## Results

### Household transmission in England

Overall, 7.7% (1849/24,060) of all TB cases notified in England between 2010 and 2012 shared a house with at least one other TB case.

**Fig. 2:**
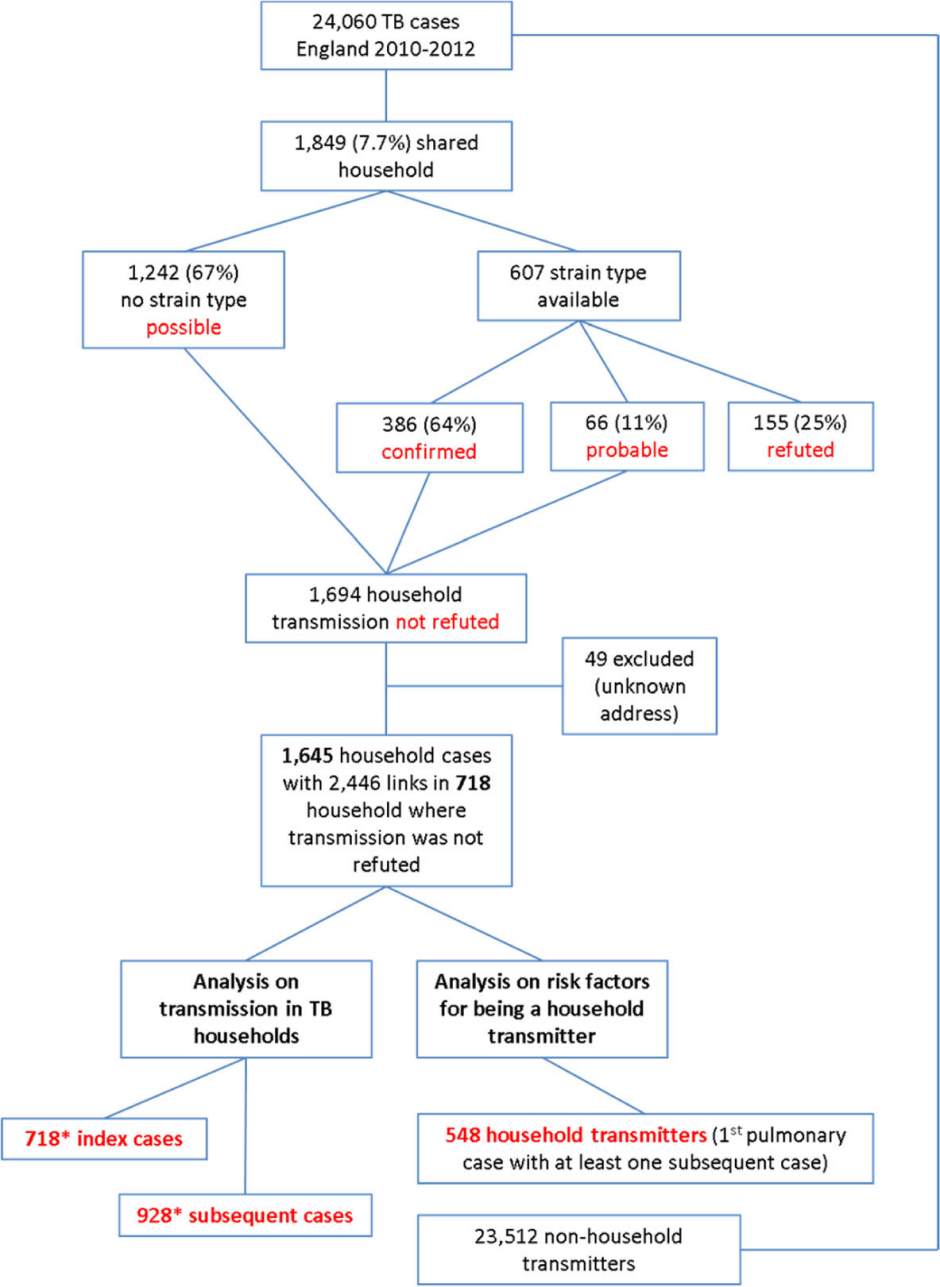
Flow chart of cases included in study. *One case lives in two households and is thus counted twice in the index case count

ST data showed that transmission was confirmed for 21% (386), probable for 3.6% (66) and refuted for 8% (155), and ST was unavailable for one or both (possible household transmission) for 67% (1242) of these cases. For those with ST, 64% (386) had confirmed, 11% probable (66), and 25% (155) refuted household transmission (Fig. 2).

There were 1694 cases living in TB households where transmission was not refuted, 49 of whom were excluded as we could not assign them to specific households. The 1645 cases lived in 718 TB households.

Applying the n-1 method [16] to account for the index case in households where transmission was not refuted, 3.9% ((1645 household cases- 718 households)/24,060 cases) of TB cases in England were estimated to be due to recent household transmission.

### Households where recent transmission is likely to have occurred

Of the 718 households where transmission was not refuted, 19.6% had more than two cases; 13.2% (95) had three cases, 4.6% (33) had four cases, and 1.8% (13) had five or more cases. A total of 2446 links were identified between 1645 cases, with 718 index cases and 928 subsequent cases.

Seventy-five percent (538/715) of index cases in households and 66.8% (603/903) of subsequent cases with known site of disease had pulmonary TB, the remainder had extra-pulmonary TB only. The majority of index cases were adults (86.5%), while almost 40% of subsequent cases were children (Table 1). Additionally, 30.7% of index and 46.6% of subsequent cases were born in the UK, the most common country of birth.

**Table 1:** Characteristics of index and subsequent cases in households where transmission was not refuted

	Index case (*n* = 718)	Subsequent cases (*n* = 928)
Sex		
Male	358/715 (50.1%)	481/924 (52.1%)
Age (years)		
0–14	97/718 (13.5%)	364/928 (39.2%)
15–44	471/718 (65.6%)	442/928 (47.6%)
45–64	113/718 (15.7%)	93/928 (10.0%)
65+	37/718 (5.2%)	29/928 (3.1%)
Country of birth^a^		
UK	210/685 (30.7%)	411/882 (46.6%)
India	129/685 (18.8%)	138/882 (15.7%)
Pakistan	78/685 (11.4%)	68/882 (7.7%)
Nepal	23/685 (3.4%)	24/882 (2.7%)
Somalia	59/685 (8.6%)	72/882 (8.2%)
Site of disease		
Pulmonary	538/715 (75.2%)	603/903 (66.8%)

^a^Top five countries of birth by number of cases notified in England between 2010 and 2012

The proportion of TB cases that occurred in a household with another TB case varied by country of birth: from 11.1% (621/5790) for UK-born cases and 10.7% (131/1230) for Somali-born cases to 2.7% (22/820) for Bangladeshi-born cases. Twenty six (1.6%) household TB cases living in 15 different households had MDR-TB.

### Treatment delay by household order

Information on treatment delay (time from symptom onset to treatment start) was known for 68% (488/718) of index cases and for 55% (512/928) of subsequent cases. The median treatment delay was 65 days for index cases, decreasing to 40 days for subsequent cases (Table 2).

**Table 2:** Treatment delay (symptom onset to treatment start) for index cases and subsequent cases in TB households where dates were known

	Treatment delay
	Median days (IQR)
Index case^a^ (*n* = 488)	65 (34–124)
All subsequent cases^a^ (*n* = 512)	40 (18–85)
Subsequent symptomatic^b^ cases (*n* = 110)	61 (31–177)
Subsequent asymptomatic^b^ cases (*n* = 402)	37 (16–70)

^a^Where treatment delay is known (dates to calculate treatment delay are not available for all cases)

^b^At index cases’ first contact with healthcare

**Fig. 3:**
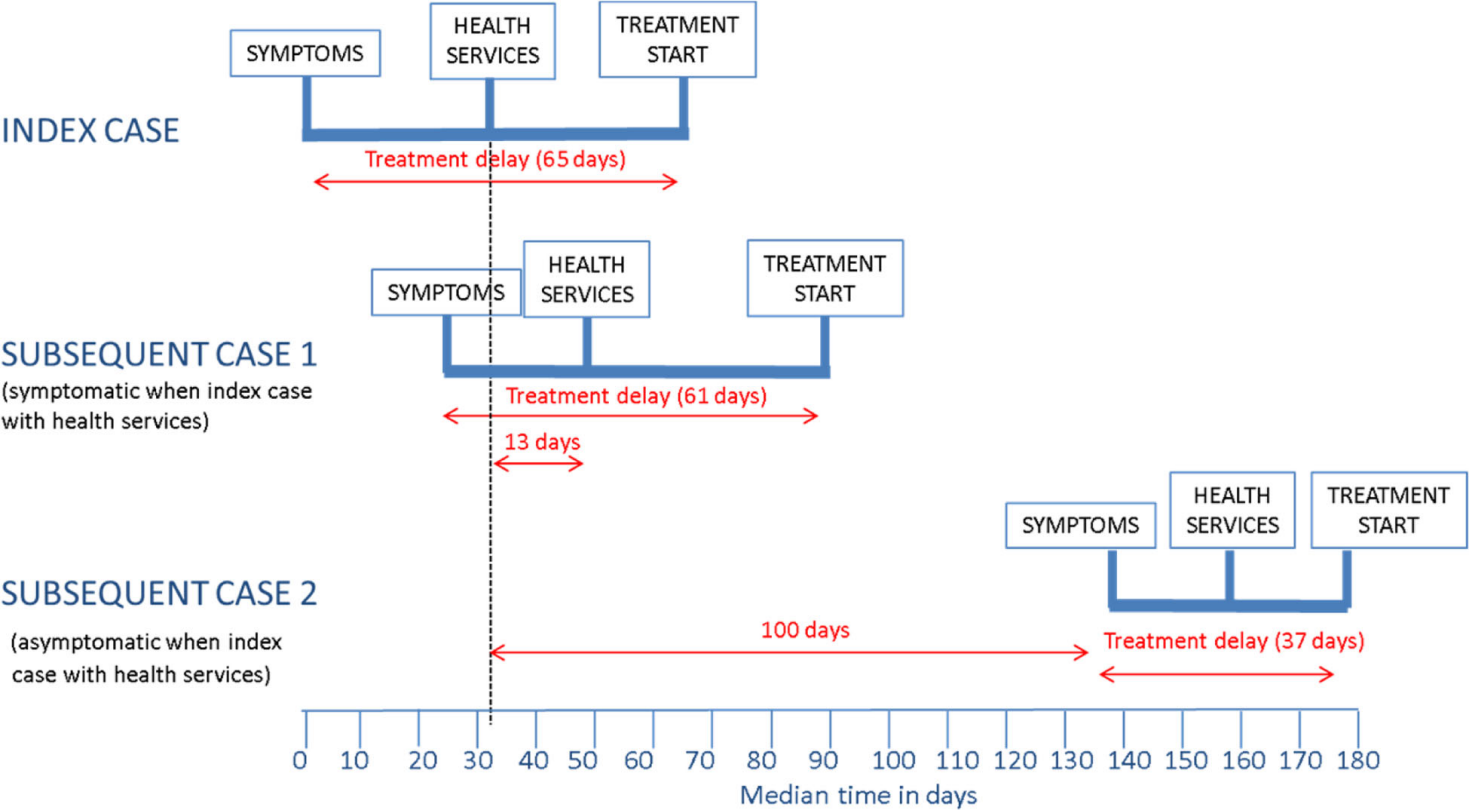
Diagram showing timing of key events in patient pathway for index cases and subsequent cases stratified according to whether they were symptomatic when the index case first made contact with healthcare

Seventy-eight percent (402/512) of subsequent household TB cases were not yet symptomatic when the index case made contact with healthcare. Subsequent cases already symptomatic had a similar treatment delay (median of 61 days) to the index case, while those who were not yet symptomatic had shorter delays (median of 37 days) (Table 2, Fig. 3).

Symptomatic subsequent cases had a median delay of 13 days (IQR, 4–40) from the index case’s first contact with healthcare until the subsequent case’s contact with healthcare, and a further 14 days before treatment started, resulting in a median delay of 29 days (IQR, 9–64) from the index case’s first contact with healthcare.

Asymptomatic subsequent cases took a median time of 100 days (IQR, 44–214) to develop symptoms from first contact of the index case with healthcare. From the time of developing symptoms, subsequent cases interacted with healthcare within a median of 16 days (IQR, 1–37) and started treatment in a median time of 37 days (IQR, 16–70).

### Risk factors for being a transmitter within a household

A total of 548 transmitters (first pulmonary case in household with at least one subsequent case) were identified. Thirteen percent (69 cases) were children (< 15 years), including 21 aged five or less. In all age groups, transmitters were more likely to be reported as smear and culture positive compared to all non-household transmitters (Table 3).

**Table 3:** Culture and smear status by age group in household transmitters and all other cases

	Household transmitters		All other cases (non-household transmitters)	
	Culture positive	Smear positive	Culture positive	Smear positive
	n (%)	n (%)	n (%)	n (%)
Age				
0–14	29 (42.0)	16 (23.2)	265 (25.0)	58 (5.5)
15–24	120 (84.5)	79 (55.6)	2422 (68.6)	741 (21.0)
25–44	201 (88.5)	132 (58.1)	6806 (62.7)	1568 (14.5)
45–64	77 (92.8)	53 (63.9)	2732 (55.7)	765 (15.6)
65+	26 (96.3)	15 (55.6)	1846 (58.2)	456 (14.4)
Total	453 (92.7)	295 (53.8)	14,071 (59.8)	3588 (15.3)

Risk factors associated with recent household transmission were identified by performing univariable and multivariable analysis. We compared 548 transmitters and 23,512 TB cases (Fig. 2) who were non-household transmitters in our model, which included sex, age, ethnicity in the UK-born, country of birth and lineage of the strain (Table 4). Those aged under 25 years were more likely to be a transmitter compared to those aged 25–44 years (age 0–14: aOR, 2.56 (CI, 1.58–4.15); age 15–24: aOR, 1.47 (CI, 1.14–1.91)). However, when household transmitters were compared with all other household cases only, the odds of being a transmitter was lowest for those aged 1–14 years (OR, 0.26; 95% CI, 0.19–0.35) compared to the reference group of 25–44 year olds (data not shown). Those born in the UK with a Black African (aOR, 2.26; CI, 1.15–4.44), Indian (aOR, 2.08; CI, 1.17–3.69) or Pakistani (aOR, 2.76; CI, 1.65–4.62) ethnicity, and those born in Somalia (aOR, 1.74; CI, 1.10–2.76) or Romania (aOR, 3.47; CI, 1.73–6.96), were more likely to be household transmitters compared to those born in India. Cases with the Euro-American lineage (aOR, 1.68) and Central Asian strain (aOR, 1.58) were more likely to be transmitters compared to those with East African Indian strains, and there was no evidence of a difference in comparison with Beijing strains.

**Table 4:** Univariable and multivariable analysis identifying factors associated with household transmitters

	Household Transmitters		All other cases		Univariable analysis			Multivariable analysis		
	n	%	n	%	OR	CI	*P* value	aOR	CI	*P* value
Sex										
Male	292	53.5	13,375	57.0	0.87	0.73–1.03	0.097	0.89	0.72–1.10	0.273
Age group										
0–14	69	12.6	1059	4.5	3.11	2.36–4.11	<0.0001	2.56	1.58–4.15	<0.0001
15–24	142	25.9	3529	15.0	1.93	1.55–2.38		1.47	1.14–1.91	0.003
25–44	227	41.4	10,848	46.1	1.00			1.00		
45–64	83	15.2	4903	20.9	0.81	0.63–1.04		1.06	0.79–1.41	0.719
65+	27	4.9	3172	13.5	0.41	0.27–0.61		0.51	0.32–0.82	0.005
Country of birth/Ethnicity										
White UK born	74	14.2	3308	15.0	1.40	1.02–1.93	0.037	1.46	0.97–2.20	0.069
Black Caribbean UK born	6	1.2	268	1.2	1.40	0.61–3.25	0.427	1.21	0.51–2.89	0.666
Black African UK born	27	5.2	335	1.5	5.06	3.22–7.93	<0.0001	2.26	1.15–4.44	0.018
Indian UK born	21	4.0	551	2.5	2.39	1.47–3.90	<0.0001	2.08	1.17–3.69	0.013
Pakistani UK born	35	6.7	655	3.0	3.35	2.23–5.03	<0.0001	2.76	1.65–4.62	<0.0001
Bangladeshi UK born	5	1.0	110	0.5	2.85	1.13–7.18	0.026	2.75	0.94–8.03	0.064
Other UK born	13	2.5	290	1.3	2.81	1.55–5.11	0.001	1.86	0.85–4.09	0.121
India	80	15.4	5019	22.7	1.00			1.00		
Pakistan	54	10.4	2939	13.3	1.15	0.81–1.63	0.424	1.17	0.78–1.77	0.452
Somalia	44	8.5	1186	5.4	2.33	1.60–3.38	<0.0001	1.74	1.10–2.76	0.019
Bangladesh	7	1.3	813	3.7	0.54	0.25–1.17	0.120	0.81	0.34–1.90	0.620
Nepal	15	2.9	583	2.6	1.61	0.92–2.82	0.093	1.26	0.67–2.35	0.471
Nigeria	12	2.3	521	2.4	1.45	0.78–2.67	0.240	1.25	0.60–2.61	0.557
Zimbabwe	14	2.7	456	2.1	1.93	1.08–3.43	0.026	1.69	0.87–3.25	0.119
Philippines	2	0.4	355	1.6	0.35	0.09–1.44	0.147	0.30	0.04–2.19	0.233
Kenya	5	1.0	302	1.4	1.04	0.42–2.58	0.935	1.25	0.49–3.19	0.635
Sri Lanka	4	0.8	286	1.3	0.88	0.32–2.41	0.800	0.54	0.13–2.23	0.393
Afghanistan	6	1.2	269	1.2	1.40	0.60–3.24	0.432	1.21	0.47–3.08	0.693
Eritrea	8	1.5	250	1.1	2.01	0.96–4.20	0.064	1.66	0.74–3.73	0.221
Romania	11	2.1	163	0.7	4.23	2.21–8.10	<0.0001	3.47	1.73–6.96	<0.0001
Poland	6	1.2	165	0.8	2.28	0.98–5.31	0.055	2.12	0.88–5.13	0.094
Other country	72	13.8	3296	14.9	1.37	0.99–1.89	0.055	1.21	0.82–1.80	0.329
Lineage										
Euro-American	173	42.4	4494	37.2	2.31	1.55–3.43	<0.0001	1.68	1.09–2.59	0.018
CAS	117	28.7	3535	29.3	1.98	1.32–2.99	0.001	1.58	1.03–2.45	0.038
EAI	29	7.1	1738	14.4	1.00			1.00		
Beijing	23	5.6	634	5.3	2.17	1.25–3.79	0.006	1.66	0.93–2.97	0.086
*M. africanum*	2	0.5	107	0.9	1.12	0.26–4.76	0.878	0.85	0.19–3.72	0.830
*M. bovis*	1	0.3	49	0.4	1.22	0.16–9.16	0.845	1.21	0.16–9.27	0.852
*M. microti*	0	0.0	1	0.0	1.00			1.00		
Multiple classifications	19	4.7	315	2.6	3.61	2.00–6.53	<0.0001	2.65	1.41–4.97	0.002
Unknown	44	10.8	1198	9.9	2.20	1.37–3.54	0.001	1.70	1.03–2.81	0.038

*CAS* Central Asian strain, *EAI* East African Indian

A sensitivity analysis showed that results were not significantly different when defining recent household transmission as ‘confirmed and probable transmission events’ or ‘confirmed, probable and possible transmission events’ (Additional file 1: Table S1).

## Discussion

To our knowledge, this is the first study to combine molecular ST and epidemiological data on links between cases to estimate the contribution of recent household transmission of TB at a national level. Our analysis of TB occurring over a 3-year period identified confirmed, probable and possible household transmission events, enabled us to describe diagnostic delay for index and subsequent cases in households, and allowed us to investigate risk factors for being a household transmitter.

We found that almost 8% of TB cases in England occurred within a household with another TB case, allowing us to estimate that 4% of TB cases were due to recent transmission within households. Given the lack of a gold standard for identifying recent transmission of TB, it is not possible to compare this to robust estimates of the overall proportion of TB cases attributable to TB transmission in England. In England, 58.4% of cases cluster by MIRU-VNTR, so the maximum proportion of cases that could be estimated to be due to recent transmission using this method is 47%, which would be an overestimate [1].

While only 30% of household index cases were born in the UK (similar to all notified TB cases) [1], we noted that 48% of subsequent household cases were UK-born (some likely to be children of non-UK-born parents). Whilst we might expect UK-born cases to be due to recent transmission in the UK, the fact that 10% of UK-born TB cases lived in a household with two or more cases suggests that earlier diagnosis and improved contact tracing could have an important impact in this group.

This study provides important insights into the characteristics of cases that transmit TB within households. After adjusting for age, sex and lineage, transmitters were more likely to be from Black-African, Indian and Pakistani ethnic groups in the UK-born, or from Somalia or Romania compared with India. Possible reasons these cases are more likely to transmit could include that they live in larger households, with different composition, socioeconomic status and overcrowding. The presence in the household of long-term visitors from high incidence countries, lack of awareness about TB, stigma resulting in obstacles to access healthcare/treatment, and lack of willingness to disclose information about contacts could also play a part. There is evidence that stigma, fear of discrimination, social exclusion and deportation, and other sociocultural factors can be barriers to Somali patients accessing TB services and disclosing their TB status to facilitate contact tracing [17–20], but less research has been performed on obstacles faced by other communities. Interestingly, we found that UK-born cases of South Asian ethnicity were more likely to be household transmitters compared to those born in South Asia. One possible explanation is that both patients and clinicians may be less likely to suspect TB in UK-born cases compared to migrants, resulting in a longer diagnostic delay, increased periods of infectivity and more transmission. It would be interesting to explore the reasons for this differential level of transmission based on ethnicity and country of birth in future studies.

The majority of index cases were adults (86%) and almost 40% of subsequent cases were children. Surprisingly, given that children are usually considered to be less infectious than adults [21], we found children to be the index case in 14% of households. In the risk factor analysis, children had the highest odds of being a transmitter compared to other age groups, when comparing household transmitters with all other TB cases. This may in part reflect the fact that children always live in a household with other people, whereas adults may live alone, and when household transmitters were compared with all other household cases only, the odds of being a transmitter was lowest for children compared to 25–44 year olds. In some instances, all household cases could have been infected outside the household, but the child developed symptoms faster. However, as child transmitters were more likely to be smear and culture positive (and thereby have the potential to transmit) compared to non-transmitters, we should not assume that adults within the household are always the source case. Exposure to infectious TB may have occurred within the community, with the age of the child influencing both their risk of exposure [22] and the likelihood of transmitting to others.

There is significant delay from symptom onset to treatment start in pulmonary TB cases, with median delay of 72 days in 2015 [1]. We found that treatment delay was similar for index cases and subsequent household cases already symptomatic at the time of identification of the index. Household contact tracing should lead to earlier detection of incident active TB cases in the household. It is therefore of concern that there was a median delay of 29 days from the identification of the index case until subsequent symptomatic cases started treatment. For subsequent household cases who were asymptomatic when the household index case was identified, there was a reduction in the delay from symptom onset to treatment, likely due to contact tracing and to the provision of ‘inform and advise’ information. However, a median delay of more than 1 month (37 days) demonstrates there is potential to further reduce this delay.

Our study has many strengths; we used national data, ST results for all culture confirmed cases and complete data on addresses of cases over a 3-year period. However, there are limitations too; we have assumed that, if two cases occur in the same household and have indistinguishable MIRU-VNTRs, this represents recent household transmission. It may occasionally reflect transmission from a non-household source case to members of the same household, which may have occurred during a visit or in another setting. This study only collected data on active TB cases occurring within a household within a 3-year time period; we were not able to collect data on latent TB infection. We recognise that due to the long and variable incubation period for reactivation, this study may therefore underestimate household transmission. A high proportion of TB cases in the UK are not culture confirmed, and therefore do not have ST results. Household cases with ‘refuted’ transmission according to ST may be misclassified with differences occurring due to microevolution or technical limitations with the laboratory processes. Furthermore, some dates recorded in the surveillance system for symptom onset and first contact with healthcare may not be accurate and result in misclassification of household order.

## Conclusions

Our study has a number of implications for household contact tracing in England. The fact that 25% of TB cases in households with ST results had discordant strains shows that households with multiple TB cases do not necessarily represent household transmission. ST data should be used to identify refuted cases of household transmission, and to inform the need for source contact tracing outside the household, especially for children. It is interesting to note that one-quarter of index cases in households had extra-pulmonary TB only; if the new NICE guidelines [13] are followed, and household contact tracing around extra-pulmonary TB cases is not performed, there will be a missed opportunity to identify subsequent cases in 25% of households, potentially leading to further transmission from undiagnosed pulmonary cases. The considerable delay before subsequent cases in households are diagnosed and start treatment suggests improvements could be made in speed of identifying contacts and conducting screening. Our finding that no lineage of TB was associated with recent household transmission, with no increased transmissibility in the Beijing lineage compared to others, suggests that the lineage need not impact contact tracing efforts; a study from Canada supports this finding [23]. Finally, we found household transmission was more common for cases born in Somalia or Romania and certain UK-born ethnic groups, suggesting that targeted interventions, such as raising awareness within these high-risk communities, and overcoming barriers to effective contact tracing could have an impact on reducing transmission of TB in England.

The Collaborative Tuberculosis Strategy for England 2015–2020 cites contact tracing as a key activity for TB control requiring strengthening [24]. Actions could include more home-based visits, improved awareness and education, and should consider the sociocultural aspects of health-seeking behaviour. Our study suggests such improvements could reduce not only the number and rate of TB in UK and non-UK-born populations, but also the transmission of TB within England.

## Additional file

Additional file 1: Table S1. Sensitivity analysis comparing multivariable analysis of household transmitters versus all other TB cases when possible transmitters (without strain typing results) were included or not. (DOCX 24 kb)

## Abbreviations

aORs: Adjusted odds ratios
HL: Household epidemiological link
IQR: Inter quartile range
MIRU-VNTR: Mycobacterial Interspersed Repetitive Unit-Variable Number Tandem Repeats
ML: Molecular links
NICE: National Institute for Health and Care Excellence
ST: Strain typing
TB: Tuberculosis
