# Supplementary material for: Recent household transmission of tuberculosis in England, 2010–2012: retrospective national cohort study combining epidemiological and molecular strain typing data
Source: BMC Med. 2017 Jun 13;15:105. doi: 10.1186/s12916-017-0864-y (PMC5469076; doi:10.1186/s12916-017-0864-y)
Supplement: Additional file 1: Table S1. — Sensitivity analysis comparing multivariable analysis of household transmitters versus all other TB cases when possible transmitters (without strain typing results) were included or not. (DOCX 24 kb) [file 12916_2017_864_MOESM1_ESM.docx]

Additional file 1

**Table S1: Sensitivity analysis comparing multivariable analysis of household transmitters vs all other TB cases when possible transmitters (without strain typing results) were included or not**

|  |  | Multivariable analysis (transmitter (pulmonary confirmed+probable+possible) vs all others | | | Multivariable analysis (transmitter (pulmonary confirmed+probable) vs all others (including possible)) | | |
| --- | --- | --- | --- | --- | --- | --- | --- |
|  |  | **aOR** | **CI** | **P value** | **aOR** | **CI** | **P value** |
| Sex | Male | 0.89 | 0.72-1.10 | 0.273 | 0.94 | 0.69-1.29 | 0.710 |
| Age | 0-14 | 2.56 | 1.58-4.15 | <0.0001 | 2.91 | 1.41-5.99 | 0.004 |
|  | 15-24 | 1.47 | 1.14-1.91 | 0.003 | 1.80 | 1.22-2.66 | 0.003 |
|  | 25-44 | 1.00 |  |  | 1.00 |  |  |
|  | 45-64 | 1.06 | 0.79-1.41 | 0.719 | 1.25 | 0.81-1.94 | 0.314 |
|  | 65+ | 0.51 | 0.32-0.82 | 0.005 | 0.38 | 0.17-0.85 | 0.019 |
| Country of birth/Ethnicity | White UK-born | 1.46 | 0.97-2.20 | 0.069 | 2.17 | 1.20-3.95 | 0.011 |
|  | Black Caribbean UK-born | 1.21 | 0.51-2.89 | 0.666 | 1.46 | 0.42-5.03 | 0.549 |
|  | Black African UK-born | 2.26 | 1.15-4.44 | 0.018 | 1.19 | 0.33-4.27 | 0.787 |
|  | Indian UK-born | 2.08 | 1.17-3.69 | 0.013 | 3.08 | 1.43-6.63 | 0.004 |
|  | Pakistani UK-born | 2.76 | 1.65-4.62 | <0.0001 | 3.35 | 1.60-6.98 | 0.001 |
|  | Bangladeshi UK-born | 2.75 | 0.94-8.03 | 0.064 | 1.57 | 0.20-12.18 | 0.665 |
|  | Other UK-born | 1.86 | 0.85-4.09 | 0.121 | 1.53 | 0.44-5.37 | 0.502 |
|  | India | 1.00 |  |  | 1.00 | 0.00-0.00 |  |
|  | Pakistan | 1.17 | 0.78-1.77 | 0.452 | 0.85 | 0.42-1.72 | 0.648 |
|  | Somalia | 1.74 | 1.10-2.76 | 0.019 | 2.00 | 0.99-4.02 | 0.053 |
|  | Bangladesh | 0.81 | 0.34-1.90 | 0.620 | 1.00 | 0.00-0.00 |  |
|  | Nepal | 1.26 | 0.67-2.35 | 0.471 | 1.49 | 0.62-3.58 | 0.378 |
|  | Nigeria | 1.25 | 0.60-2.61 | 0.557 | 0.76 | 0.17-3.31 | 0.71 |
|  | Zimbabwe | 1.69 | 0.87-3.25 | 0.119 | 1.78 | 0.65-4.88 | 0.265 |
|  | Philippines | 0.30 | 0.04-2.19 | 0.233 | 1.16 | 0.15-9.27 | 0.889 |
|  | Kenya | 1.25 | 0.49-3.19 | 0.635 | 1.88 | 0.55-6.39 | 0.314 |
|  | Sri Lanka | 0.54 | 0.13-2.23 | 0.393 | 1.00 | 0.00-0.00 |  |
|  | Afghanistan | 1.21 | 0.47-3.08 | 0.693 | 1.06 | 0.24-4.61 | 0.936 |
|  | Eritrea | 1.66 | 0.74-3.73 | 0.221 | 2.24 | 0.75-6.64 | 0.148 |
|  | Romania | 3.47 | 1.73-6.96 | <0.0001 | 0.73 | 0.10-5.57 | 0.762 |
|  | Poland | 2.12 | 0.88-5.13 | 0.094 | 0.90 | 0.12-6.83 | 0.916 |
|  | Other country | 1.21 | 0.82-1.80 | 0.329 | 1.26 | 0.68-2.32 | 0.46 |
| Lineage | Euro-American | 1.68 | 1.09-2.59 | 0.018 | 2.80 | 1.21-6.45 | 0.016 |
|  | CAS | 1.58 | 1.03-2.45 | 0.038 | 2.99 | 1.30-6.88 | 0.01 |
|  | EAI | 1.00 |  |  | 1.00 | 0.00-0.00 |  |
|  | Beijing | 1.66 | 0.93-2.97 | 0.086 | 3.70 | 1.39-9.81 | 0.009 |
|  | *M.africanum* | 0.85 | 0.19-3.72 | 0.830 | 1.00 | 0.00-0.00 |  |
|  | *M.bovis* | 1.21 | 0.16-9.27 | 0.852 | 4.35 | 0.50-37.71 | 0.182 |
|  | *M.microti* | 1.00 |  |  | 1.00 | 0.00-0.00 |  |
|  | Multiple classifications | 2.65 | 1.41-4.97 | 0.002 | 2.42 | 0.68-8.61 | 0.172 |
|  | Unknown | 1.70 | 1.03-2.81 | 0.038 | 3.07 | 1.23-7.69 | 0.016 |
